# Monogenic Immune Diseases Provide Insights Into the Mechanisms and Treatment of Chronic Graft-Versus-Host Disease

**DOI:** 10.3389/fimmu.2020.574569

**Published:** 2021-02-04

**Authors:** Jacob Rozmus

**Affiliations:** ^1^ Division of Pediatric Hematology, Oncology & BMT, Department of Pediatrics, BC Children’s Hospital, University of British Columbia, Vancouver, BC, Canada; ^2^ Michael Cuccione Childhood Cancer Research Program, BC Children’s Hospital Research Institute, Vancouver, BC, Canada

**Keywords:** primary immunodeficiency diseases, chronic graft-versus-host disease, hematopoietic stem cell transplantation, inflammation, T cell, B cell

## Abstract

Chronic graft-versus-host disease (GvHD) has become a leading cause of morbidity and mortality following allogeneic hematopoietic stem cell transplantation (HSCT) and can burden patients with devastating and lifelong health effects. Our understanding of the pathogenic mechanisms underlying chronic GvHD remains incomplete and this lack of understanding is reflected by lack of clear therapeutic approaches to steroid refractory disease. Observations predominantly from mouse models and human correlative studies currently support a three phase model for the initiation and development of chronic GvHD: 1) early inflammation and tissue damage triggers the innate immune system. This leads to inflammatory cytokine/chemokine patterns that recruit effector immune cell populations; 2) chronic inflammation causes the loss of central and peripheral tolerance mechanisms leading to emergence of pathogenic B and T cell populations that promote autoimmune and alloimmune reactions; 3) the dysregulated immunity causes altered macrophage polarization, aberrant tissue repair leading to scarring and end organ fibrosis. This model has led to the evaluation of many new therapies aimed at limiting inflammation, targeting dysregulated signaling pathways and restoring tolerance mechanisms. However, chronic GvHD is a multisystem disease with complex clinical phenotypes and it remains unclear as to which cluster of patients will respond best to specific therapeutic strategies. However, it is possible to gain novel insights from immune-related monogenic diseases. These diseases either share common clinical manifestations, replicate steps from the three phase chronic GvHD model or serve as surrogates for perfectly targeted drugs being investigated in chronic GvHD therapy. In this review, we will summarize the evidence from these monogenic immune related diseases that provide insight into pathogenic pathways in chronic GvHD, rationales for current therapies and novel directions for future drug discovery.

## Chronic Graft-Versus-Host Disease

Chronic graft-versus-host disease (cGvHD) is now the leading cause of morbidity and mortality post-hematopoietic stem cell transplantation (1, 2). cGvHD is a pleomorphic syndrome that resembles autoimmune and other immunologic disorders that occurs between 3 and 15 months after HCT. Chronic GvHD can affect almost any organ including skin, liver, eyes, mouth, lungs, gastrointestinal tract, neuromuscular system, or genitourinary tract. The spectrum of disease manifestations and diagnostic criteria were updated in 2014 after the second National Institutes of Health (NIH) Consensus Conference on cGvHD (3). The rates of cGvHD depend on several variables and can range from as low as 6% in matched sibling cord blood transplants to as high as 65% in matched unrelated donor (MUD) peripheral blood stem cell (PBSC) transplants (4).

Our understanding of the pathophysiology of cGvHD has improved over the last decade to the point where there is now a well-accepted three phase model of cGvHD development supported by mouse models, correlative clinical studies and clinical trials (5). The three phases are: 1) acute inflammation and tissue injury trigger inflammatory cytokine/chemokine patterns, mediated through the innate immune system, that recruit effector immune cell populations; 2) chronic inflammation causes a loss of tolerance that disrupts the homeostasis of the adaptive immune system leading to the emergence of pathogenic B and T cell populations; 3) the dysregulated immune response causes altered macrophage polarization causing an aberrant tissue repair mechanism leading to excessive end organ fibrosis and scarring.

Despite these insights, clinicians continue to struggle to identify the optimal therapy for patients with cGvHD who do not respond to front-line corticosteroids or patients who cannot be successfully weaned off corticosteroids.

## Why Study Rare Diseases?

Rare inherited monogenic diseases affecting innate and adaptive immunity provide a unique opportunity to understand the role of specific genes, molecules, pathways and cell types in our immune system (6). Unfortunately, in the past these rare diseases were often considered medical outliers and neglected compared to more polygenic, multifactorial common disorders. However, they all operate under the same biological principles and these rare diseases are actually much simpler pathologically then common diseases. These human models demonstrate the function of a particular gene in an otherwise controlled experiment of nature, in which everything else is identical except for the one single factor which is the root cause of the resulting disease phenotype. Better understanding rare diseases not only directly benefits those patients afflicted but the recognition of a molecular defect can lead to potential therapies. Mutations that alter the level of activity of gene products can be thought of as surrogates for perfectly targeted drugs (7).

Their study provides the means to better understand complex acquired diseases in a number of ways:

1. An acquired disease may have a specific phenotype that is specifically missing in an inherited disease due to the absence of a key molecule. By pharmacologically inhibiting this factor you may eliminate the phenotype, therefore the inherited disease informs a potential new target.
2. An acquired disease may have a specific phenotype that mimics that seen in an monogenic disease associated with altered function (gain or loss of function) of a key molecule or cell type; inherited disease again provides potential new target or supports the addition of a key factor into treatment, such as adding an agonist or cell type. The emergence of cellular therapies has given clinicians the ability to treat disease with a wide variety of manipulated cell types in addition to the well-established therapy of hematopoietic stem cell transplant.
3. A new targeted therapy may be trialed based on mouse models or human correlative clinical studies of a specific disease and there is a corresponding monogenic disease involving that factor; the rare disease may provide insights into unintended consequences of targeting that factor in other biological pathways.

With these principles in mind, the purpose of this review is to use our evolving understanding of monogenic immune disorders to provide a rationale for previous and ongoing therapies in cGvHD and potentially provide new avenues for intervention based on the pathophysiology of cGvHD (**Table 1**).

**Table 1:** Potential therapies targeting the pathophysiology of each phase of chronic graft-versus-host disease.

cGvHD Phase	Phase 1	Phase 2	Phase 3
*Pathophysiology*	Acute inflammation and tissue injury activates the innate immune system including the complement system leading to the recruitment of pathogenic cell populations	Loss of tolerance mechanisms disrupts the homeostasis of the adaptive immune system	Aberrant tissue repair mechanism leading to excessive end organ fibrosis and scarring
*Relevant monogenic diseases*	*Primary immune deficiencies (PID) with inflammatory bowel disease (IBD)-like pathology* *MyD88 and IRAK-4 deficiencies* *Complement deficiencies*	*Autoimmune polyendocrinopathy-candidiasis-ectodermal dystrophy (APECED)* *Immune dysregulation, polyendocrinopathy, enteropathy, X-linked (IPEX) syndrome* *LPS-responsive beige-like anchor protein (LRBA) deficiency* *Cytotoxic T lymphocyte antigen-4 (CTLA-4) haploinsufficiency* *Autosomal dominant hyper-IgE syndrome (AD-HIES)* *IL-12/23 receptor beta 1 (IL-12/23Rβ1) and IL-12/23 cytokine p40 subunit deficiency* *Glut1 deficiency* *Leptin deficiency* *Wiskott-Aldrich syndrome (WAS)* *B cell activating factor (BAFF) receptor deficiency* *Gain-of-function mutations in PIK3CD* *X-linked agammaglobulinemia (XLA)*	*Matrix metalloproteinase-2 (MMP-2) deficiency* *Stiff skin syndrome (SSS)*
*Potential therapies*	***Treatment of gut dysbiosis:*** selective use of antibiotics, pre- and probiotics, fecal microbiota transplantation (FMT) ***TLR inhibition:*** MyD88 and IRAK-4 inhibitors Statins Hydroxychloroquine ***Complement inhibitors:*** Eculizumab [anti-C5 monoclonal antibody (mAb)], narsoplimab (IgG-4 mAb against MASP-2), and coversin (C5 inhibitor)	***Thymic transplantation:*** Medullary thymic epithelial cells (mTECs) ***Reduce donor T cell migration:*** Sphingosine 1-phosphate receptor (S1PR) agonists ***Increase number and function of regulatory T cells (Tregs):*** Rapamycin (mTOR inhibitor), abatacept (CTLA4-Ig), hydroxychloroquine, low dose IL-2, extracorporeal photopheresis, and infusion of donor-specific or third party Tregs. ***Targeting the Th17 subset:*** ROCK2 inhibitor KD025, tocilizumab (mAb against IL-6 receptor), ustekinumab (IL-12 and IL-23 antagonist), pirfenidone ***Targeting T cell metabolic reprogramming:*** Inhibition of glycolysis, leptin, glutamate-oxaloacetate transaminase (GOT1), and glutaminase (GLS) ***Targeting B cell mediated autoimmunity:*** Rituximab (anti-CD20 mAb), belimumab (anti-BAFF mAb), Syk inhibitors, PI3K inhibitors, ibrutinib (BTK inhibitor)	***Target activation of collagen-producing fibroblasts and myofibroblasts:*** Imatinib mesylate (tyrosine kinase inhibitor), sonidegib (sonic hedgehog pathway inhibitor) ***Increase levels of dermal MMP-2:*** Narrowband ultraviolet-B light therapy ***Integrin inhibition:*** Natalizumab (mAb against α4-integrin) and vedolizumab (α4β7 inhibitor)

The bolded text in italics describes the mechanism/class of medications that addresses the pathophysiology of that phase of cGvHD.

## Similarities Between Chronic GvHD and Primary Immune Disorders

Chronic GvHD is fundamentally a disorder of immune regulation. A successful HCT requires: 1) reconstitution of normal innate and adaptive cellular immune responses to infectious pathogens and the 2) induction of immune tolerance to non-self antigens and in the case of malignant disease, while preserving the graft-versus-tumor effect.

The persistent alloreactivity in cGvHD is driven step-wise by increased expression of host-derived molecules that result from tissue damage. This leads to the expansion of pathogenic T and B cell populations that escape tolerance and are allowed to persist due to the failure of suppressive regulatory mechanisms. This promotes chronic inflammation that triggers aberrant repair mechanisms. Therefore, this review will focus on primary immunodeficiencies associated with defects in intrinsic or innate immunity, autoimmunity and dysregulation of lymphocyte homeostasis.

## Defects Involving the Innate Immune System

The intestinal epithelium, an integral component of innate immunity, is altered in a number of ways during the HCT process; complications of the primary disease, gastrointestinal infections, conditioning chemotherapy and radiation cause direct intestinal damage and the use of broad-spectrum antibiotics and varied diets disrupt gut microbiota. Affected cell types include: 1) intestinal stem cells, impairing epithelial regeneration, 2) intestinal epithelial cells, which comprises barrier function, 3) Paneth cells, leading to decreased secretion of antimicrobial peptides, and 4) goblet cells, which depletes the mucus barrier. The cumulative effect is a dysbiosis associated with decreased commensal bacterial function and diversity, increased gut permeability and bacterial translocation leading to increased local inflammation that disrupts immune homeostasis (8).

Many primary immune deficiencies are associated with microbial dysbiosis, which manifests clinically as inflammatory bowel disease (IBD)-like pathology (9) and may alter the clinical phenotype of a common genetic susceptibility. Underlying pathogenic mechanisms include the absence of secretory IgA which normally promotes the clearance of antigens and pathogenic bacteria from the gut microbiota (10) and increased translocation of lipopolysaccharide (LPS) (11). Therapies such as the selective use of antibiotics, prebiotics, probiotics and fecal microbiota transplantation, aimed at restoring the gut microbiota may prove beneficial in cGvHD and are actively being investigated (12).

Early inflammation in patients post-HCT is triggered by the activation of innate pattern-recognition receptors (PRRs) such as Toll-like receptors (TLRs) and nucleotide oligomerization domain (NOD)-like receptors (NLRs) on host antigen presenting cells (APCs) by viral and bacterial components and endogenous dangerous molecules termed danger-associated molecular patterns (DAMPs). These signals are released due to endothelial and epithelial cell damage in the GI tract caused by underlying disease, infection and transplant conditioning. TLR signaling in APCs such as dendritic cells enhance antigen endocytosis and autophagy and augments the assembly of key antigen transport and processing systems (13). In turn, activated host and donor APCs stimulate donor T cells either directly through donor T-cell receptors that recognize minor histocompatibility antigens, foreign MHC molecules and allogeneic peptides or indirectly through the release of pro-inflammatory cytokines and chemokines such as IL-1β, IL-6, IL-8, IL-10, IL-12, IL-21, IL-23, TGFβ and TNFα. Compared with non-GVHD patients after HSCT and healthy donor controls, TLR4-mediated NF-κB signaling-related genes including TLR4, NF-κB, IL-6 and intercellular adhesion molecules 1 (ICAM-1) were significantly increased in patients with cutaneous cGVHD (14).

In innate immune cells such as dendritic cells, MyD88 is the critical adaptor molecule that bridges TLRs to the IRAK family of kinases, which in turn stimulate a signaling cascade that results in NF-κβ activation (15, 16). Germline MyD88 and IRAK-4 deficiencies predispose patients to recurrent life-threatening bacterial diseases, such as invasive pneumococcal disease in particular, with weak signs of inflammation (17).

There is evidence that TLR signaling contributes to the early activation of APCs and priming of donor T cells. TLR inhibition can be achieved either by blocking the binding of agonists to corresponding TLRs or inhibiting the intracellular signaling of the TLR pathways.

The use of a novel MyD88 inhibitor, TJ-M2010-5 in a fully MHC-mismatched murine model inhibited the LPS-stimulated activation of dendritic cells and the priming of donor allogeneic T cell proliferation (18). Administration of the inhibitor ameliorated the inflammatory environment, increased tissue repair in GvHD target organs and suppressed lethal GvHD. Administration of an IRAK-4 inhibitor also ameliorated GvHD in a mouse model of allo-SCT (19). In this model, MyD88 in donor T cells was not essential for graft-versus-leukemia (GvL) effects. There are other well-established medications that have been repurposed in cGvHD because of their effects on TLR signaling including statins, which decrease TLR4 expression and downstream signaling (20, 21) and hydroxychloroquine, an inhibitor of TLR9 signaling (22). Any strategy to block TLR signaling pathways incur significant risk, particularly during post-transplant immune reconstitution as TLR-mediated inflammation functions to protect the host against infection.

Another major part of the innate immune response, the complement system, is also implicated in cGvHD. The complement system is composed of a number of diverse signaling pathways that causes specific plasma proteins to react with one another to generate: 1) activated complement proteins that bind pathogens triggering opsonization by phagocytes, 2) fragments of some complement proteins that serve as chemoattractants and 3) membrane attack complexes that damage bacteria by creating pores in the outer bacterial membrane. All the pathways merge at the proteolytic cleavage of C3 to generate a larger fragment, C3b, that marks a target for opsonization, and a small one, C3a, which serves as an anaphylatoxin which triggers the release of inflammatory mediators from nearby cells. Subsequent cleavage of another complement protein, C5, results in C5a, which is also an anaphylatoxin and chemotactic factor, and C5b which initiates formation of the membrane attack complex (23). The anaphylatoxins C3a and C5a exert their biological function by binding to their cognate G protein-coupled receptors C3aR and C5aR on cells of the innate and adaptive immune system.

Human C3 deficiency is associated with impairments in dendritic cell maturation suggesting complement activation could play a role in the dendritic cell regulation of GvHD in first acute phase of inflammation (24). The generation of C3 and C5 complement proteins during complement activation has previously been implicated in the pathogenesis of GvHD. Expression of C3aR and C5aR on donor T cells is essential for GVHD development after HCT (25). Reduced GvHD in C3-deficient mice is associated with decreased donor Th1/Th17 differentiation (26). C3aR/C5aR-mediated signaling directly induces secretion of IFN-γ and IL-2 from T cells driving Th1/Th17 differentiation and suppressing Treg generation (27, 28). C3aR/C5aR signaling suppresses lethal mitophagy in dendritic cells after HCT. Blockade of C3aR/C5aR activation significantly enhanced mitophagy in recipient dendritic cells which correlated with improved GvHD outcomes and the studies also showed that treatment with C3aR/C5aR antagonists effectively separated GvHD and GvL responses making it a promising therapeutic approach for GvHD treatment especially in malignant diseases dependent on the GVL effect (29, 30).

In the post-allo-HCT setting, complement inhibitors such as eculizumab, (anti-C5 monoclonal antibody), narsoplimab (IgG-4 monoclonal antibody that inhibits the effector enzyme MASP-2 of the lectin complement pathway), and coversin (C5 inhibitor) have already been used in the treatment of transplant-associated thrombotic microangiopathy (TA-TMA) (31–33). It is possible that these therapies could be used as prophylaxis for cGvHD.

Targeting these innate pathways appears promising as they would ideally interrupt the early inflammatory cascade underlying cGvHD development while preserving the GvL effect.

A potential issue would be the timing of these interventions as they target the early stages of cGvHD which are difficult to appreciate clinically and maybe better served as prophylactic agents against GvHD or used in specific patients early post-HCT with predictive biomarkers (34).

## T Cell Immune Dysregulation

Ongoing damage to epithelial and connective tissue releases DAMPs that activate cells of the innate immune system such as dendritic cells triggering the release of IFNα, IL-1β, TNFα and IL-6. This inflammatory cytokine profile induces Th1/Th17 differentiation and subsequent recruitment to the injured tissue. They are activated by APCs and continue the cycle of tissue damage. Dysfunctional thymic negative selection frees alloreactive T cells targeting host antigens that continually feed this vicious cycle leading to chronic tissue inflammation. This part of the review will focus on two key elements of this pathological process: 1) loss of thymic negative selection and 2) skewing of T cell repertoire toward Th1/Th17 lineages at the expense of regulatory T cells. Both of these biological processes have parallels with monogenic immune disorders that provide insights into pathology and the basis for existing and potential new therapies.

### Loss of T Cell Thymic Selection

Early after HCT, mature donor alloreactive T cells transferred with the allograft are activated by host APCs and mediate direct tissue destruction. In particular, thymic epithelial cells are damaged leading to release of self-reactive T cells. Severe histopathological damage to the thymus is a feature of aGvHD and plays a prominent role in the second phase of cGvHD. Using murine models of allogeneic HCT it has previously been shown that donor T cells can damage primary lymphoid tissue including the thymus. Thymic aGvHD impaired the compartment of medullary thymic epithelial cells (mTEC) that express the autoimmune regulator (AIRE) (35, 36). Loss of AIRE+ mTEC led to a failure to clonally delete self-reactive T cells. This is likely caused by the decreased heterogeneity of tissue specific auto-antigens from cGVHD target organs presented by thymic mTEC cells in order to select functional but tolerant T cells. Accordingly, donor-derived T cells possessing cGvHD antigen reactivity escape deletion and expand. This loss of thymic negative selection is further exacerbated by the physiologic process of age-related thymic atrophy/involution (37). This pool of self-reactive T cells is under constant homeostatic pressure to expand due to overall lymphopenia in GvHD caused by the dysfunction of the peripheral niches essential for the survival of naïve T cells (38).

The AIRE gene is mutated in autoimmune polyendocrinopathy-candidiasis-ectodermal dystrophy (APECED), a rare monogenic recessive disorder characterized by a variety of autoimmune diseases that target endocrine organs, liver, intestine and skin (39). This is caused by immune reactions against an assortment of autoantigens (40). Murine studies suggest that AIRE promotes ectopic transcription of self-antigens in mTECs and therefore important for negative selection of autoreactive T cells or in the case post-HCT, alloreactive T cells (41). All patients with APECED also have neutralizing antibodies against type I interferons and they are present before the development of autoimmune conditions (42). Antibodies to IFNα have also been recognized as an autoantibody that develops after allogeneic BMT in association with cGvHD (43, 44). Lastly, APECED patients also have a decrease in the regulatory T cell population (45) similar to patients with cGvHD.

It is unclear whether thymic transplantation, which has been used successfully in the treatment of differentiative thymic disorder related to FOXN1 mutations (46), would alter the process of negative selection by the thymus. The transplantation of recipient-type thymus at 4 weeks post-BMT in an established chronic GvHD model prevented the development of cGvHD and increased survival (47). Perhaps in the future it will be possible to generate mTECs from recipients, for example through the use of induced pluripotent stem cells, prior to HCT that can be used as prophylactic treatment post-HCT to support normal T cell development (48).

Another potential therapeutic target is preventing the trafficking of alloreactive T cells to the thymus in the early stages post-HCT thereby limiting damage to the thymus thus preserving tolerance mechanisms. This same principle could be applied to the trafficking of pathogenic T cells to target organs and ideally preserving trafficking of regulatory T cells. Sphingosine 1-phosphate (S1P) is a sphingosine containing lipid intermediate obtained from ceramide that plays a key role in lymphocyte migration through concentration gradients and binding and activation of G-protein-coupled receptors known as S1P receptors (S1PR1) (49). It has been shown that prophylactic, not therapeutic, administration of a S1PR agonist reduced donor T cell migration to the host thymus, thus significantly attenuating thymic aGvHD in murine model of unconditioned recipients of haploidentical donor T cells (50). This approach was successfully used in a patient with severe CNS GVHD (51).

### Skewing of T-Cell Repertoire During Chronic GvHD Development

The acute inflammation of the first phase of cGvHD creates an environment that favors excessive pro-inflammatory Th17 cells over regulatory T cells that suppress inflammation. The development of cGvHD has been shown to be associated with a dynamic imbalance that favors the production, expansion, and persistence of effector T cells, in particular Th17 cells driven by BCL2 expression over CD4 regulatory T cells (52). Patients with active cGvHD had a significantly lower frequency of circulating T follicular helper cells (cTFH) compared with patients without cGvHD. This was associated with higher CXCL13 plasma levels suggesting increased homing of TFH to secondary lymphoid organs. The cTFH phenotype was skewed toward a highly activated profile with predominance of Th2/Th17 subsets and demonstrated increased functional ability to promote B cell immunoglobulin secretion and maturation (53). Again their survival was preferentially promoted by BCL-2.

The creation of this immune imbalance in patients with active cGvHD lends itself to potential therapies either previously used in PIDs associated with T cell disorders or provides information about which gene products should be targeted to create an effect that mimics the PID phenotype; if cGvHD is associated with elevated Th17 cells then we should target affected proteins/pathways in monogenic diseases associated with loss of Th17 cells.

The potential impact of current therapies and new avenues of treatment are discussed in the context of known PIDs with abnormal T cell homeostasis.

### Strategies to Increase the Number of Regulatory T Cells

The prototypical genetic autoimmune disease involving Tregs is Immune dysregulation, polyendocrinopathy, enteropathy, X-linked (IPEX) syndrome which is caused by mutations in the FOXP3 gene and characterized by markedly decreased or absent FOXP3+ Tregs (54). Many other primary immunodeficiencies with prevailing lymphoproliferation, such as LPS-responsive beige-like anchor protein (LRBA) deficiency and cytotoxic T lymphocyte antigen-4 (CTLA-4) haploinsufficiency, are also associated with decreased or dysregulation of Tregs (55, 56).

Rapamycin is a small molecule inhibitor of mechanistic target of rapamycin (mTOR) that selectively inhibits effector T cell proliferation while sparing rapamycin-resistant Treg cells thereby supporting the relevant expansion and function of Treg cells (57). Rapamycin has significant clinical benefits in patients with IPEX syndrome (58) and has previously shown efficacy as a cGvHD therapy (59, 60).

Abatacept (CTLA4-Ig) is a fusion protein consisting of an IgG1 Fc domain fused to the CTLA-4 extracellular domain that has successfully been used to control autoimmune inflammation and interstitial lung disease in patients with CTLA-4 haploinsufficiency and LRBA deficiency (61, 62). In a phase I clinical trial, abatacept resulted in a clinical response in 44% of patients with steroid-refractory cGvHD with both decreased prednisone use and T cell PD-1 expression in responders (63).

Inhibition of lysosomal degradation *via* chloroquine/hydroxychloroquine rescued CTLA4 expression in LRBA deficient cells *in vitro* and improved lymphoproliferative lung pathology in a patient with LRBA mutation *in vivo* (64) and long term outcome of patients with LRBA deficiency (65). A phase II trial of hydroxychloroquine in patients with steroid-resistant or steroid-dependent cGvHD resulted in a 53% response rate and all responders tolerated a >50% reduction in their steroid dose while receiving hydroxychloroquine (66).

IL-2 is a critical cytokine for the maintenance and function of FOXP3+ Treg cells. High CD25 expression confers to Treg cells the ability to respond to low doses of IL-2, whereas effector T cells require higher IL-2 concentrations to support their proliferation. Patients with Wiskott-Aldrich syndrome who received low dose IL-2 therapy had statistically significant increase in platelet counts, a trend toward higher T, B, and NK cell numbers and higher T regulatory cell percentages (67). Low dose IL-2 has been shown to provide durable clinical improvement in active cGvHD and extended therapy is well-tolerated (68).

It still remains controversial as to whether extracorporeal photopheresis (ECP) has a clinically significant effect on the number and function of Tregs in cGvHD (69–71).

Multiple studies have demonstrated that developing mixed chimerism post-HCT in non-malignant disease is associated with a lower incidence of aGvHD and cGvHD and among patients with mixed chimerism, cGvHD is associated with a more frequent evolution toward complete chimerism (72). The proportion of Treg cells is increased in patients with mixed chimerism after SCT and acts to suppress the alloreactive immune response (73). In non-malignant diseases, especially those undergoing reduced intensity conditioning resulting in dynamic chimera states, interventions to increase Tregs may stabilize mixed chimerism and lead to lower rates of cGvHD.

Lastly, the Infusion of donor-specific or third-party regulatory T cells have been tested in patients with steroid-refractory or dependent cGvHD. A phase I trial utilizing donor derived Tregs enriched by CD25+ immunomagnetic selection from a non-mobilized peripheral blood apheresis product and purified by high speed flow cytometry demonstrated feasibility, safety and tolerability with encouraging preliminary clinical responses with a single infusion of cells (74). Patients have also been treated with umbilical cord blood derived regulatory T cells (75).

### Targeting the Th17 Subset

Autosomal dominant hyper-IgE syndrome (AD-HIES), formerly known as Job syndrome, caused by loss of function mutations in STAT3, is associated with impaired Th17 development (76). Th17 cell development is directed by multiple cytokines, including IL-1β, IL-6, TGF-β, IL-21 and IL-23 which leads to activation of the transcription factors STAT3 and interferon regulatory factor 4 and subsequent expression of retinoic acid-related orphan receptor (ROR)yt. It has been shown that oral administration of the selective ROCK2 inhibitor KD025 to healthy subjects or rheumatoid arthritis patients attenuates the ability of T cells to secrete IL-17 in response to stimulation ex vivo *via* a STAT3-dependent mechanism. ROCK2 inhibition significantly diminished STAT3 phosphorylation and binding to IL-17 and IL-21 promoters and reduced interferon regulatory factor 4 and nuclear hormone RORyt protein levels in T cells derived from healthy subjects or rheumatoid arthritis patients. Simultaneously, KD025 also promoted the suppressive function of regulatory T cells through up-regulation of STAT5 phosphorylation (77). KD025 has been shown to ameliorate cGvHD in multiple murine models and inhibit the secretion of IL-21, IL-17 and interferon y along with decreasing phosphorylated STAT3 and reduced protein expression of interferon regulatory factor 4 and B-cell lymphoma (BCL6) in human peripheral blood mononuclear cells purified from active cGvHD patients (78).

IL-6 is a proinflammatory cytokine that activates the STAT3 signaling cascade and promotes Th17 differentiation. Tocilizumab, the monoclonal antibody against the IL-6 receptor, has been used to treat STAT3 gain of function disease (79). Tocilizumab appears to be a promising treatment option in advanced cGvHD but further evaluation within a phase II trial is required (80).

Inherited IL-12/23 receptor beta 1 (IL-12/23Rβ1) and IL-12/23 cytokine p40 subunit deficiency are rare primary immunodeficiencies associated with impaired generation of IL-17 producing cells (81). Anti-p40 treatment attenuated the severity of sclerodermatous cGvHD in a murine model (82). Ustekinumab, a human IL-12 and IL-23 antagonist, delivered by subcutaneous injection on day -1 and day +20 after peripheral blood mobilized hematopoietic stem transplantation from HLA-matched sibling or unrelated donors significantly improved overall survival and National Institute of Health (NIH) moderate/severe cGvHD-free, relapse-free survival (83). It has not yet been tested in patients with existing cGvHD.

Pirfenidone has been shown to inhibit IL-17A facilitated macrophage infiltration in a mouse model of cGvHD lung disease. In addition, pirfenidone significantly reduced the percentage of IL-17a-producing CD4+ T cells but did not affect the percentage of Tregs (84).

## Targeting Metabolic Reprogramming as a Potential Therapeutic Strategy

A number of dysregulated metabolic pathways have previously been identified in PIDs and in turn congenital defects in metabolism are often associated with immune defects. Targeting these pathways in cGvHD offers new avenues of potential therapy.

It has been shown that glycolysis is required for optimal function of alloantigen-activated T cells and induction of GVHD. T cells switch from fatty acid β-oxidation and pyruvate oxidation *via* the tricarboxylic (TCA) cycle to aerobic glycolysis. Inhibition of glycolysis through specifically targeting mTORC1 or PFKFB3 ameliorated GVHD in a preclinical BMT model (85).

Glut1 deficiency selectively impairs metabolism and function of thymocytes and effector CD4 T cells while sparing Treg cells (86). Allo-reactive Glut1-deficient T cells have dramatically decreased ability to induce lethal GvHD due to reduced IL-17 production.

Congenital deficiency of the adipocyte hormone leptin is associated with reduced numbers of circulating CD4+ T cells and impaired T cell proliferation and cytokine release (87). In contrast, increased serum leptin concentrations may contribute to T cell activation during development of cGvHD (88).

There is literature that shows that by simply inhibiting transamination in differentiating T cells, Th17 cell fate can be epigenetically redirected toward the Treg lineage. A recent study identified a compound, (aminooxy)acetic acid (AOA), that is able to reprogram differentiating Th17 cells into Foxp3-expressing iTreg cells by inhibiting the activity of glutamate-oxaloacetate transaminase (GOT1) (89). Another group were able to show that transiently inhibiting glutamine metabolism by targeting glutaminase activity lead to impaired differentiation of Th17 cells and increased Th1 and CTL effector cell function (90).

Selectively targeting metabolic pathways in order to alter the balance of TH17/Treg cells may represent a novel strategy to treat chronic GvHD.

## The Role of B Cell Mediated Autoimmunity

Chronic GvHD has many clinical, histological and serological manifestations that resemble the autoimmunity and dysgammaglobulinemia associated with primary B-cell related immunodeficiencies. Multiple lines of evidence point to an important role for B cells in the pathogenesis of cGvHD. Antibodies to both alloantigens and nonpolymorphic autoantigens are frequently associated with cGvHD (91, 92). Stimulatory antibodies to the platelet-derived growth factor (PDGF) receptor (PDGFR) are selectively found in patients with extensive cGvHD and activate the generation of reactive oxygen species which stimulates type 1 collagen gene expression suggesting a role in the development of fibrosis (93). Allogeneic HY antibodies detected at 3 months after female to male HCT predict cGvHD in humans (94). B cells facilitate autoimmunity not just by secreting host-reactive antibodies, but also by secreting proinflammatory cytokines and by presenting autoantigens to T cells (95). Conversely, an impaired ability of B cells to produce IL-10 was found in patients with active cGvHD (96). Perhaps the best evidence for B cell involvement is the success of rituximab, a chimeric anti-CD20 monoclonal antibody, in corticosteroid-free primary treatment of cGvHD (97–99).

The emergence and persistence of host-reactive B cells in cGvHD results from acquired failures in tolerance mechanisms. Central B cell tolerance is compromised in cGvHD due to altered B cell signalling that affects the negative and positive selection of B cells during development, skewing the emerging B cell repertoire towards a host or self-reactivity. Autoantibody production may also occur due to disturbed T cell- B cell interaction and regulation (100). The highest rate of autoimmune cytopenias following HCT are reported in children undergoing HCT for non-malignant indications with anti-thymocyte globulin (ATG) or alemtuzumab-containing conditioning regimens (101).

The most important driver of immature bone marrow B cell tolerance is B-cell receptor (BCR) signaling after encountering self-antigens. Developmental fate is based on strength and location of BCR engagement, the form of self-antigen and synergy with other co-receptor signals (102). It is clear there are intrinsic and extrinsic factors that can skew this process and overcome other processes that would normally remove autoreactive B cells. Two of the most critical signaling pathways that integrate with BCR signaling in B cell survival and tolerance are Toll-like receptor (TLR) and B cell-activating factor receptor (BAFFR) signaling.

TLR activation appears to contribute to both the negative and positive selection of autoreactive B cells depending on the developmental stage based on observations in PID patients. Patients who lack MyD88 or IRAK-4 exhibit defects in central and peripheral B cell tolerance, implicating TLR-dependent innate signaling pathways in negative selection of immature autoreactive B cell clones (103, 104). In contrast, there is evidence for TLR signaling promoting transitional B cell positive selection in patients with Wiskott-Aldrich syndrome (WAS). There is enhanced signaling downstream of both the BCR and TLRs in B cells from WAS patients that promotes the positive selection of autoreactive transitional B cells (105, 106).

B cell activating factor (BAFF) plays a fundamental role in the survival and differentiation of B cells (107). Its’ principal cognate receptor in early B cell development is the BAFF receptor (BAFF-R). Without BAFF-R, B-cell development is arrested at the stage of transitional B cells and the numbers of all subsequent B cell stages are severely reduced (108). Increased BAFF levels rescue low-affinity self-reactive transitional B cells by co-opting BCR signaling through phosphorylation of proximal BCR signaling components such as spleen tyrosine kinase (Syk) (109). BAFF also enhances TLR7/9 expression on B cells and TLR-mediated production of autoantibodies (110). In turn, TLR signaling promotes BAFF receptor expression creating a positive feedback loop (111). Murine models of B cell autoimmunity suggest that excess BAFF and a reduced pool of naïve B cells are both necessary to promote the survival of autoreactive B cells (112, 113). BAFF has also been shown to selectively enhance the survival of plasmablasts which would promote the subsequent production of host-reactive antibodies (114).

Chronic GvHD is associated with reduced transitional and naïve B cell counts (115), elevated levels of sBAFF (116) and Syk hyperresponsiveness in B cells.

Belimumab, a fully human monoclonal IgG1λ anti-BAFF antibody, is currently being tested as prophylaxis against chronic GvHD in a phase 1 trial (NCT03207958). Inhibition of Syk with fostamatinib in mice with established cGvHD with bronchiolitis obliterans was able to reverse disease. It also decreased the frequency of GCs and expression of the activation costimulatory molecules CD80 and CD86 in CD11c+ cells *in vivo*. Most importantly, human cGvHD B cells had increased death when treated with fostamatinib (117). Inhibiting Syk kinase activity abrogates the BCR-driven ex vivo proliferative and survival advantage of human cGvHD B cells (118).

Another example of a PID with a B cell specific break in self-tolerance are patients with gain-of-function mutations in PIK3CD, encoding the p110δ catalytic subunit of phosphoinositide 3-kinase (PI3K), who present with production of germline autoreactive IgM antibodies (119). PI3K expression has been shown to be increased in cGvHD patients (120). The effective treatment of mice with active cGvHD with PI3K-specific inhibitors support future clinical trials of approved PI3K inhibitors for cGvHD therapy in humans (121).

In humans, central B cell tolerance checkpoints are also abrogated in the absence of Bruton’s tyrosine kinase (BTK), an essential BCR signaling component (122). Patients suffering from X-linked agammaglobulinemia, caused by loss of function mutations in the BTK gene, have a severe decrease of peripheral B cells and serum immunoglobulin. B cell differentiation is severely affected at the pro- to pre-B transition but the few B cells that do develop are paradoxically enriched in autoreactive clones. The use of antileukemic drugs that inhibit Btk signaling to promote apoptosis of malignant B cells, especially in chronic lymphocytic leukemia, theoretically may also affect B cell selection by interfering with normal BCR signaling leading to the release of autoreactive B cells. Autoimmune cytopenias have been observed in patients with chronic lymphocytic leukemia treated with ibrutinib (123, 124).

Treatment of patients with active cGvHD with inadequate response to corticosteroid-containing therapies with ibrutinib, a BTK inhibitor, in a phase II clinical trial resulted in clinically meaningful responses with acceptable safety leading it to become the only FDA-approved second-line therapy for steroid-resistant cGvHD (125, 126).

Targeting Btk in cGvHD patients with ibrutinib also highlights the potential for phenotypic differences between germline presentations and the effects of an imperfect inhibitor. In addition to its critical role in B cell development, BTK is important for collagen signaling *via* the collagen receptor glycoprotein VI (GPVI) in platelets (127). Ibrutinib has been reported to increase rates of major hemorrhage through selective inhibition of platelet signaling and functions downstream of the collagen receptor GPVI and strongly affects firm platelet adhesion on von Willebrand factor (VWF) under arterial flow (128). In contrast to ibrutinib-treated subjects, patients with XLA do not bleed excessively. The risk of bleeding is attributed to off-target effects of ibrutinib on several other intracellular molecules important for platelet signaling including Tec, another kinase of the Tec family of protein-tyrosine kinases that includes Btk (129). Ibrutinib can also affect T cells due to the off-target inhibition of IL-2 inducible T cell kinase (ITK) with shares significant homology with BTK. Ibrutinib treatment in chronic lymphocytic leukemia (CLL) patients markedly increases CD4+ and CD8+ T cell numbers, decreases the Treg/CD4+ T cell ratio and reduced PD-1 and CTLA-4 expression in T cells (130). It remains unclear if the efficacy of ibrutinib in targeting B cells in cGvHD will be offset by changes in T cell populations, significant risk of bleeding and potential flares of autoimmunity.

## Fibrotic End Stages of cGvHD

Fibrosis represents the end stage of the chronic inflammation that occurs in cGvHD and once fixed is poorly amenable to any known therapies. It is thought to result from an aberrant wound-healing process driven by M2-polarized macrophages that in turn produce transforming growth factor-β (TGF-β) and platelet-derived growth factor-α (PDGF-α) leading to the activation of collagen-producing fibroblasts and myofibroblasts partly through sonic hedgehog signaling (131). The prominent role of TGF-β, PDGF-α and sonic hedgehog (SHH) in stimulating fibroblasts has led to the use of tyrosine kinase inhibitors such as imatinib mesylate and the Hedgehog pathway inhibitor sonidegib in the treatment of sclerotic cGvHD (132–134).

Even though there is a lack of a strong association between primary immune disorders and fibrosis, sclerotic or scleroderma-like changes, there are other rare monogenic diseases that have already or may provide new therapeutic avenues. Mutations in MMP2, an antifibrotic metalloproteinase, may result in scleroderma-like skin thickening (135). Patients post-HCT with low levels of plasma MMP-2 were more likely to develop sclerotic cGvHD (136). Narrowband ultraviolet-B light therapy, which is known to increase the level of dermal MMP-2 (137), has successfully been used to treat sclerotic cGvHD (138).

Mutations in fibrillin-1 cause stiff skin syndrome (SSS), an autosomal dominant congenital form of scleroderma (139). These mutations all localize to the domain in fibrillin-1 that harbours a motif needed to mediate cell-matric interactions by binding cell-surface integrins. Aggressive skin fibrosis in mouse lines harbouring analogous mutations was prevented by integrin-modulating therapies and reversed by antagonism of TGF-β (140). Perhaps there is an role for integrin inhibition in the prophylaxis or treatment of cGvHD analogous to the use of natalizumab (monoclonal antibody against α4-integrin) and vedolizumab (α4β7 inhibitor) in the treatment of steroid refractory aGvHD of the gut (141, 142).

## Concluding Remarks

Immune disorders due to single gene defects offer invaluable insights into understanding the immune dysregulation that occurs during all three phases of cGvHD development. One of the issues that clinicians continue to struggle with is the timing of interventions either as prophylaxis or treatment and the ideal therapy or combination of therapies depending on the specific clinical cGvHD phenotype. It is clear that by the time many of the clinical manifestations of cGvHD, in particular fibrotic and sclerotic changes, are evident many of the therapies targeting earlier phases of inflammation may be ineffective. This only reinforces the urgent need to develop predictive and prognostic biomarkers that properly identify earlier stages of the disease where interventions may be more effective.

It is clear the cGvHD is a heterogeneous disease with multiple pathogenic pathways operating simultaneously and superior treatments will only emerge from an improved understanding of disease mechanisms.

## Author Contributions

The author confirms being the sole contributor of this work and has approved it for publication.

## Conflict of Interest

The author declares that the research was conducted in the absence of any commercial or financial relationships that could be construed as a potential conflict of interest.

The reviewer DW declared a past co-authorship with the author JR to the handling editor.
